# Nonlinear Energy Harvesting by Piezoelectric Bionic ‘M’ Shape Generating Beam Featured in Reducing Stress Concentration

**DOI:** 10.3390/mi14051007

**Published:** 2023-05-06

**Authors:** Chao Xiong, Nan Wu, Yuncheng He, Yuan Cai, Xianming Zeng, Peichen Jin, Minyi Lai

**Affiliations:** Reserch Center for Wind Engineering and Engineering Vibration, Guangzhou University, Guangzhou 510006, China; 2112116243@e.gzhu.edu.cn (C.X.); yuncheng@gzhu.edu.cn (Y.H.); 2112016080@e.gzhu.edu.cn (Y.C.); zxm@e.gzhu.edu.cn (X.Z.); xiaojin_0424@163.com (P.J.); 1035734681@foxmail.com (M.L.)

**Keywords:** low frequency, M-type, bistable, energy barrier

## Abstract

Inspired by the flapping wings of seagulls during flight, a new low-cost, magnet-free, bistable piezoelectric energy harvester is proposed to obtain energy from low-frequency vibration and convert it into electrical energy and reduce fatigue damage caused by stress concentration. In order to optimize the power generation efficiency of this energy harvesting, finite element analysis and experimental tests were carried out. The results of finite element analysis and experimental results are in good agreement, and the superior performance in improving stress concentration of the energy harvester compared to the previous parabolic (bow-shaped) one using bistable technology was quantitatively analyzed using finite element simulation, with a maximum stress reduction of 32.34%. The experimental results showed that under optimal operating conditions, the maximum open-circuit voltage of the harvester was 11.5 V, and the maximum output power was 73 μW. These results indicate that this is a promising strategy, which provides a reference for collecting vibrational energy in low-frequency environments.

## 1. Introduction

With the continuous development and improvement of wireless sensors and low-power electronic technology, the conversion of ambient vibration energy into electrical energy for powering has been widely studied by researchers [1]. Vibration energy harvesting, as a renewable energy production method, converts vibrations caused by human movement, mechanical equipment, underwater flow, etc., into usable electrical energy. Piezoelectric [2,3,4,5,6], electromagnetic [7,8,9], electrostatic [10,11], and triboelectric [12,13] technologies are common conductive mechanisms in this field. Compared with other conduction mechanisms, piezoelectric vibration energy harvesters have received extensive attention and research because of their simple structure, high energy density, and freedom from electromagnetic interference.

Piezoelectric vibration energy harvesters mainly use the positive effect of piezoelectric sheets to convert vibration energy into electrical energy. For piezoelectric vibration energy harvesters, two electromechanical coupling modes of piezoelectric materials are mainly applied: 31 coupling mode (applying force perpendicular to the polarization direction) and 33 coupling mode (applying force parallel to the polarization direction). Using the principle of resonance, linear piezoelectric cantilever beams have been extensively studied due to their simple structure. Zhang et al. [14] proposed a rotating wind harvester that consists of multiple piezoelectric coupled cantilever beams, thus improving the harvesting efficiency. Fan et al. [15] designed an energy harvester using a roller-driven piezoelectric cantilever beam. The harvester can convert low-frequency swaying and bidirectional vibrations into high-frequency vibrations of the beam and improve the harvesting efficiency by the frequency up-conversion technique. Kim et al. [16] proposed a cantilevered piezoelectric vibration energy harvester focusing on adding proof mass. Through a combination of simulation and experimental tests, it was found that adding or reducing oscillator mass has a much greater effect on harvester performance than changing cantilever beam parameters, which can improve energy harvesting efficiency in addition to simply reducing frequency. To improve the harvesting efficiency of the energy harvester, researchers have investigated improvements such as tapered and L-shaped beams. Srinivasulu Raju et al. [17] designed a cantilever piezoelectric energy harvester with a tapered rectangular cavity and showed that the harvester had higher power than the uniform piezoelectric beam at low frequencies. Keshmiri et al. [18] proposed a novel nonlinear conical piezoelectric cantilever beam. By means of nonlinear tapered geometry and FGM design, the voltage output in the frequency domain is 19.76 times higher than that of the traditional uniformly designed cantilever beam. Xie et al. [19] developed a cylinder energy harvester made of L-shaped piezoelectric coupled beams. However, linear piezoelectric energy harvesters are only effective at a narrow frequency bandwidth near their natural frequency, which results in lower efficiency at frequencies far from the resonant frequency. In general, the stimulus frequency in the surrounding environment randomly changes within a certain bandwidth, making it impossible for the linear energy harvester to effectively capture energy beyond the resonant frequency.

To improve the operating bandwidth of the energy harvester, the researchers also introduced nonlinear [20], amplitude/frequency amplification [15], multi-resonator arrays [21], and magnetostrictive techniques [22]. The former nonlinear technology can obtain a larger bandwidth and higher amplitude response in broadband vibration, which is related to its snap-through with local high-frequency vibration and its high-power output. Among them, monostable [23], bistable [24,25,26,27], and multi-stable [28] technologies are representatives of nonlinear techniques. Bistable piezoelectric energy harvesters can be implemented by different mechanisms. The common method is to change the potential shape of the harvester by providing pre-tightening force or pre-deformation. For example, Wang et al. [29] proposed a nonlinear bi-stable energy harvester with a bulking beam. By studying the effect of axial load on the energy harvesting amplitude, it was found that the movement of the energy harvester can change from in-well motion to inter-well motion under a suitable axial load. Liu et al. [30] proposed a quasi-zero stiffness device that can use a piezoelectric bending beam to isolate vibration and collect energy and compared the energy harvesting performance of the cantilever beam energy harvester, indicating that the device can obtain higher output power and lower operating frequency. Chen et al. [31] proposed an M-shaped buckled beam bistable energy harvester, and experimental and numerical results showed that the proposed energy harvester could easily maintain large-amplitude inter-well oscillations and offer a wider operation bandwidth in a lower frequency range. Similarly, Derakhshani et al. [32] used experiments, and Pan et al. [33] used theoretical methods to study the dynamic characteristics and energy capture efficiency of bistable piezoelectric energy harvesters.

Bionic designs have recently been widely explored for the development of piezoelectric energy harvesters [34,35]. Qian et al. designed a piezoelectric energy harvesting system that can collect broadband vibrations by observing the rapid shape transition of the Venus flytrap [36]. Inspired by the parasitic relationship in plants, Fu et al. proposed a host-parasite vibration harvester, which is designed to scavenge random low-frequency vibrations by incorporating bi-stability and frequency up-conversion [37]. Inspired by the flight mechanism of dipteran, Zhou et al. [38] proposed a novel bionic-dipteran energy harvester (BDEH) to collect vibration energy at an ultralow frequency and with low-excitation acceleration. Inspired by the microstructure of sponges, Zhang et al. [39] designed a piezoelectric composite energy harvester with a significant improvement in vibration energy harvesting due to the well-distributed stress of the piezoelectric element. It is possible to convert the energy of vibrations at low frequencies and low amplitudes in the environment into electrical energy.

In conclusion, it is a feasible scheme to improve energy capture efficiency based on nonlinear technology. In this work, inspired by the flapping wings of seagulls during flight, a new low-cost, magnet-free, bistable piezoelectric energy harvester is proposed to obtain energy from low-frequency vibration and convert it into electrical energy and reduce fatigue damage caused by stress concentration. In order to optimize the power generation efficiency of this energy harvesting, finite element analysis and experimental tests were carried out. This research work can provide a new approach for capturing low-frequency environmental vibration energy based on nonlinear technology.

The remainder of the article is organized as follows. Section 2 explains the design of an “M” type power generation beam of energy harvester, mathematical model, and FEM optimization design. Section 3 describes the ability of the model to improve stress concentration. Section 4 describes the prototype fabrication of the energy harvester, the experimental process, and the output performance under different conditions. Finally, the conclusions are drawn in Section 5.

## 2. Design and Modeling

### 2.1. Design of “M” Type Power Generation Beam of Energy Harvester

**Figure 1:**
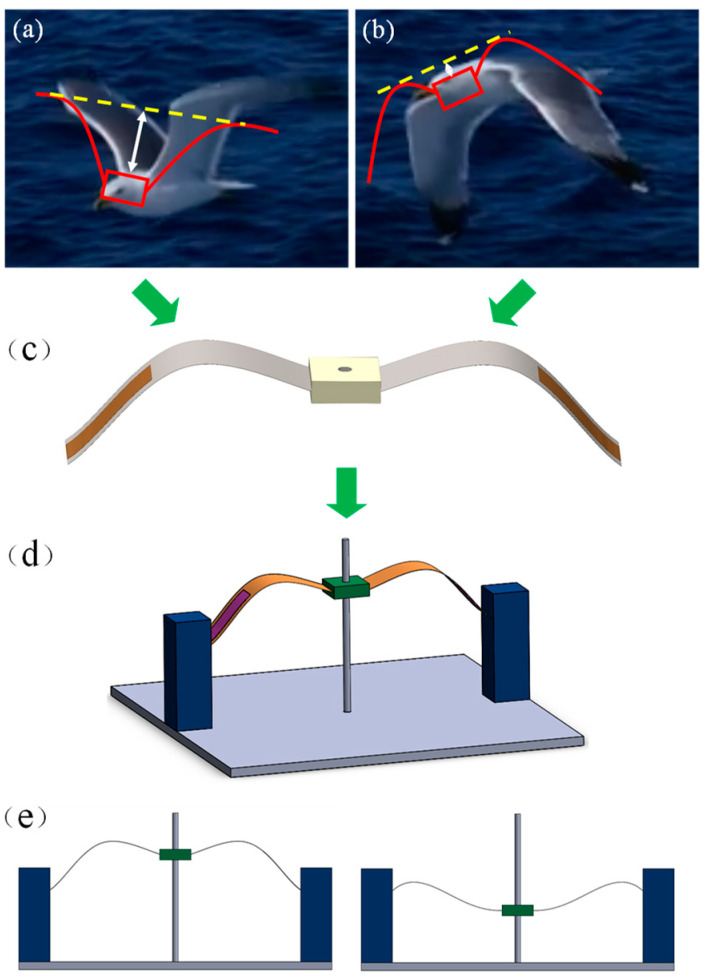
Design of the proposed bio-inspired bistable piezoelectric energy harvester (**a**,**b**) Seagull fluttering up and down in two stages of flight; (**c**) Bio-inspired power generation beam; (**d**) Bio-inspired bistable piezoelectric energy harvester device; (**e**) Bistable Phenomenon.

A good example of effective external force obtained by low-frequency nonlinear motion in nature is the flight of seagulls [40]. When flying, large birds such as seagulls mainly generate lift and thrust by flapping their wings up and down. However, the frequency and amplitude of their flaps vary at different times, as shown in Figure 1a,b. The frequency of seagull flight is relatively low and is determined by the wingspan; the wingbeat frequency of seagulls is about 5 Hz, with the lowest being 2–4 Hz [41]. During the flapping movement of the seagull’s wings, the wing chord direction also undergoes torsional deformation, and the twisting angle of the wing chord gradually increases with the spreading distance, and the torsion amplitude at the wing tip is the largest, which allows the seagull to obtain enough lift while maintaining a low fluttering frequency [42]. The rapid transition of the seagull flapping up and down is called the snap-through phenomenon. The rapid snap-through motion between two steady states causes local high-frequency vibrations due to the sudden release of energy. Sudden snap-through of nonlinear bistable structures is often associated with a large energy release, resulting in a large energy output required for vibrational energy harvesting.

Inspired by the flapping of the wings up and down during the flight of seagulls, a low-cost, magnet-free, bistable piezoelectric energy harvester is proposed to obtain energy from low-frequency vibrations and convert it into electrical energy while reducing stress concentration. The main structure of the bio-inspired bistable piezoelectric energy harvester (BBPEH) is a bio-inspired bistable beam with piezoelectric layers attached to the upper and lower surfaces, as shown in Figure 1c. The power generation part of the energy harvester consists of two power generation beams embedded in the proof mass, with PVDF piezoelectric sheets attached to the upper and lower surfaces of the power generation beams. The proof mass can slide up and down along the steel column fixed to the base, limiting its side-to-side sway. In addition, the other two ends of the power generation beam are connected to the base by embedding two square columns, thus forming a complete energy harvester device, as shown in Figure 1d. The distance between the two square columns causes a bionic pre-deformation of the energy harvester power generation beam similar to the shape of a gull wing. The two stages of a seagull fluttering up and down correspond to the upper and lower steady-states of the bio-inspired bistable piezoelectric energy harvester shown in Figure 1e.

### 2.2. Mechanical Model

**Figure 2:**
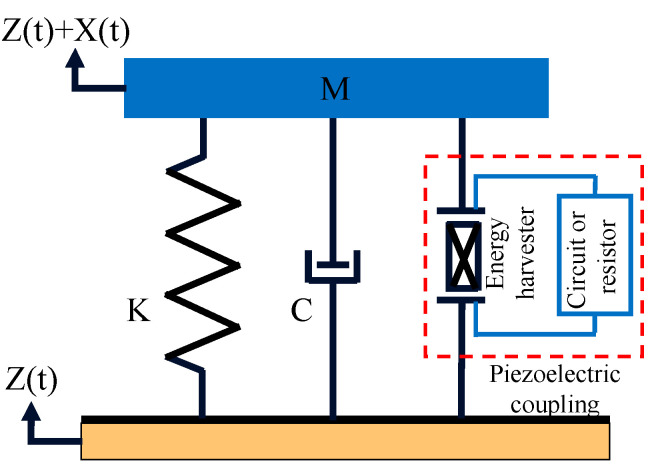
The model of mass bar spring of the bio-inspired bistable piezoelectric energy harvester.

According to the characteristics of the proposed bio-inspired bistable piezoelectric energy harvester, a lumped mass-spring model of BBPEH was established, as shown in Figure 2. Considering the influence of gravity, according to Newton’s second law and Kirchhoff’s law, the dynamic control equation of the piezoelectric spring-mass system can be written as [43]:

(1) $$\left\{\begin{array}{l} MX^{\prime \prime }+CX^{\prime }+2K(1-\frac{l}{\sqrt{X^{2}+l^{2}}})X+Mg+\Theta V=MZ^{\prime \prime }\\ C_{P}V^{\prime }+\frac{V}{R}-\Theta V^{\prime }=0 \end{array}\right.$$

where *M* is the mass of the piezoelectric vibrator (proof mass), *X* is the amplitude of the generator beam, *K* is the equivalent stiffness, *C* is the damping coefficient, *l* is the length of the generator beam, *g* is the gravitational acceleration constant, *Z*(t) is the displacement of the external vibration source as a function of time, $MZ^{\prime \prime }=A\sin(2\pi \omega t)$ (where *A* and ω are the external excitation amplitude and frequency, respectively.), *V* is the output voltage of the piezoelectric sheet, Θ is electromechanical coupling coefficient, and *Cp* is the equivalent capacitance.

Introducing the parameter ξ, Equation (1) can be rewritten as Equation (2), where $\omega =\sqrt{\frac{K}{M}}$, $\xi =\frac{C}{2\sqrt{KM}}$

(2) $$\left\{\begin{array}{l} X^{\prime \prime }+2\xi \omega X^{\prime }+\omega^{2}(1-\frac{l}{\sqrt{X^{2}+l^{2}}})X+\frac{\Theta V}{M}+g=A\sin(2\pi \omega t)/M\\ C_{P}V^{\prime }+\frac{V}{R}-\Theta V^{\prime }=0 \end{array}\right.$$

The state equation for the motion of the generation beam as a captive energy device is Equation (2). Based on the above mathematical model, the motion state of the beam can be described and predicted.

### 2.3. FEM Optimization Design

To analyze and optimize the structure of the bio-inspired bistable piezoelectric energy harvester, we conduct the finite element analysis (FEA) using COMSOL Multiphysics 5.5. By introducing parameters h and m to change the main structure of the energy harvester, we analyzed and optimized its optimal parameters. Here, h is the vertical distance between the highest point of the curved beam and the proof mass, and m is the mass of the proof mass. Using built-in solvers of the eigen frequency and frequency domain, the resonance frequency of the energy harvester and frequency domain voltage are calculated under different parameter combinations.

**Figure 3:**
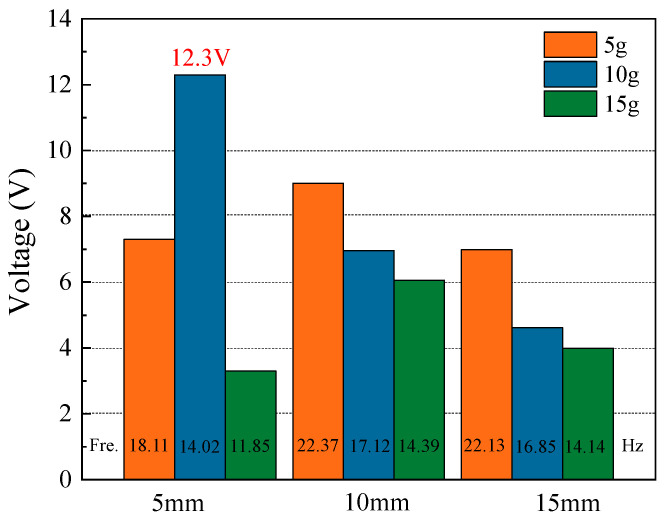
The open-circuit voltage of the energy harvester at the first-order resonant frequency for different combinations of parameters.

As shown in Figure 3, the open-circuit voltage of the energy harvester at the first resonant frequency under different parameter combinations is shown. It is obvious from Figure 3 that the resonance frequency of the energy harvester decreases with m when the parameter h remains unchanged. When h = 10/15 mm, the open-circuit voltage decreases as the mass of proof mass increases. However, when h = 5 mm, the open-circuit voltage first increases and then decreases with the mass of the proof mass increasing. Therefore, when h = 5 mm and m = 10 g, it is the optimal size of the energy harvester.

**Figure 4:**
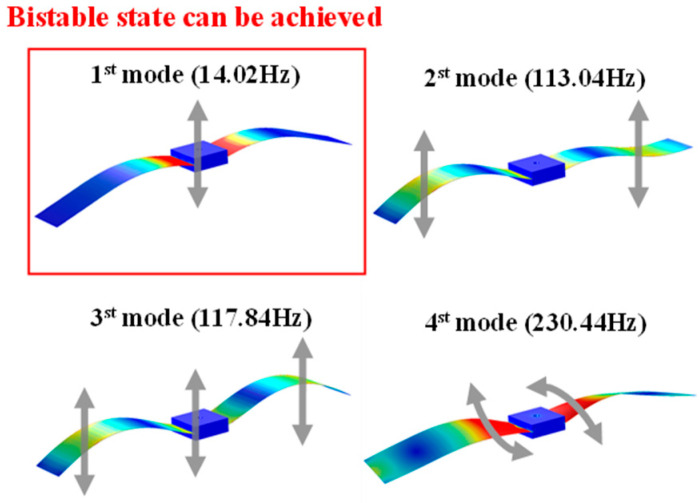
Vibration mode shapes in terms of the mode order (1st to 4th) in the FEA simulation.

Figure 4 shows the order of modes (1 to 4) of the vibration mode shapes of an energy harvester at optimal size. As can be seen from Figure 4, the first-order mode is a bending motion with a natural frequency of 14.02 Hz. The higher vibration modes corresponding to the 2nd, 3rd, and 4th harmonics exhibit asymmetric bending, symmetrical bending, and torsional motions with natural frequencies of 113.02 Hz, 117.84 Hz, and 230.44 Hz, respectively. Considering the vibration frequency and the piezoelectric patch characteristics of low natural frequency, energy is easily obtained by bending deformation, and only the first-order vibration mode is useful for bio-inspired bistable energy harvesters.

## 3. Relief of Stress Concentration

It is learned from previous literature that lots of designs of energy harvesters based on bistable technology (including generator beam and generator shell) are the parabolic form. A concentrated force is applied through the intermediate position to achieve bistability, and the stress concentration mainly occurs at the application of the force. However, the energy harvester proposed in this paper is quite different from previous designs. Firstly, the pre-deformed beam generated is not a traditional parabolic shape but a bio-inspired cambered beam based on seagull wing flapping. Secondly, when the sudden snap-through occurs in the proposed energy harvester, the stress of the pre-deformed beam will change continuously as the proof mass moves up and down. This is more conducive to relieving stress concentration compared to energy harvesters based on previous bistable technology.

**Figure 5:**
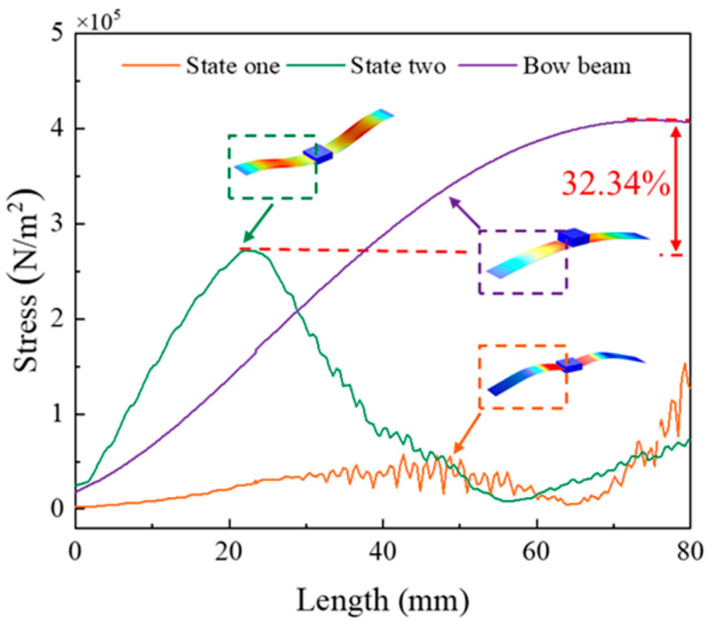
Stress profile of bio-inspired bistable energy harvester and bowed bistable harvester power generation beam.

Previous papers have only qualitatively analyzed the location of stress concentration but have not quantitatively analyzed the ability of energy harvesters to improve stress concentration compared to traditional designs. However, in this paper, the stress values of the parabolic beam of the bow energy harvester and the bionic curved beam of the energy harvester proposed in this paper at each point under the snap-through are analyzed by finite element analysis, as shown in Figure 5.

From Figure 5, it can be clearly observed that stress is concentrated at the middle position for the parabolic beam, while the bionic curved beam varies within a certain range. Compared to the parabolic beam, the maximum stress value of the bionic curved beam is reduced by 32.34%. Therefore, the energy harvester proposed in this paper can effectively improve the problem of stress concentration and extend its fatigue life.

## 4. Experiments and Analysis

### 4.1. Prototype Fabrication and Experimental Setup

**Figure 6:**
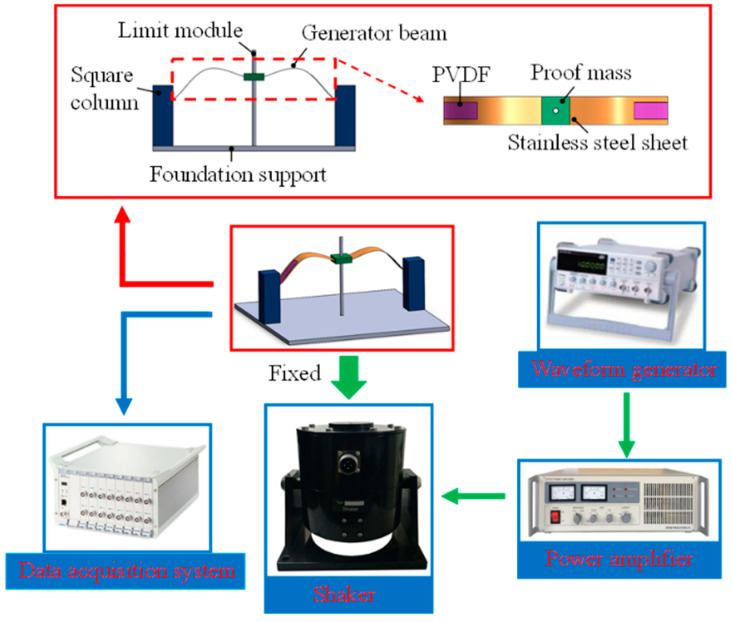
Schematic diagram of equipment.

A prototype was fabricated for experimental verification of the proposed bio-inspired bistable piezoelectric energy harvester, as shown in Figure 6. The power generation part of the energy harvester is composed of two power generation beams embedded in the middle of the proof mass, and the upper and lower surfaces of the power generation beams are equipped with PVDF piezoelectric film (IPS-17020; China Zhikang Technology Co., Ltd., Beijing, China). The power generation beam is a 301 stainless steel sheet with a thickness of 0.1 mm and a width of 20 mm. The proof mass is a 10 g resin block made of polymer methyl methacrylate (PMMA) material, which can slide up and down along the steel column fixed to the base, limiting its left and right shaking and wiping industrial petroleum jelly on the steel column to reduce frictional resistance with it. In addition, the other two ends of the generator beam are connected to the base by embedding two square columns, thus forming a complete energy harvester device. The distance between the two square columns causes a bionic pre-deformation of the energy harvester power generation beam similar to the shape of a gull wing.

The main equipment involved in the experiment are shown in Figure 6. The environmental excitation of the prototype is provided by an SA-JZ020 shaker (made by Wuxi Shiao Technology Co., Ltd. (Wuxi, China)), and the bottom plate of the model is fixed to the top rod of the vibration exciter with screws. The input signal is generated by the SA-SG030 signal generator and adjusted in amplitude by the power amplifier (SA-PA080), then fed to the shaker, and the displacement of the power beam is measured using a laser displacement meter (HG-C1200, Panasonic).

### 4.2. Output Performance of the Energy Harvester

**Figure 7:**
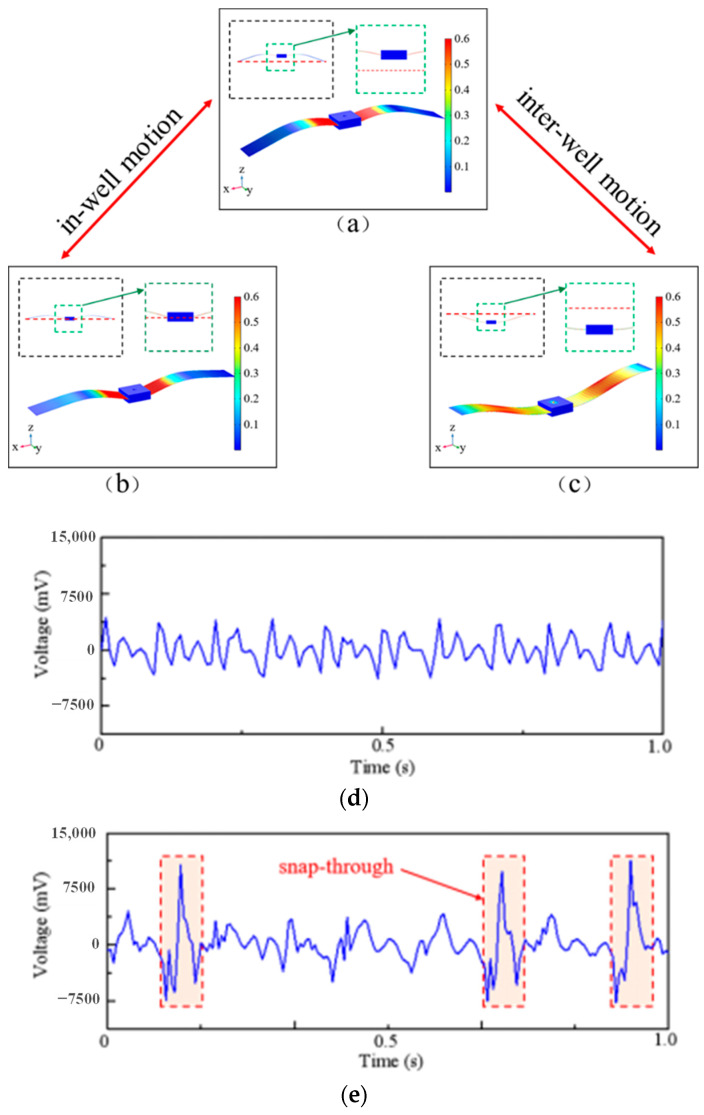
Description of the motion of the power-generating beam. When the power-generating beam is moving between (**a**,**b**), then it is in-well motion. When the power-generating beam breaks through the state of (**b**), it moves between (**a**,**c**), then it is inter-well motion. (**d**,**e**) show the output voltage of in-well motion and inter-well motion, respectively.

The flapping wing bionics energy harvester is a bistable energy capture structure. As shown in Figure 7a,c, the energy harvester has two stable states. When the external excitation is insufficient to make the power generation beam pass through the potential energy well, the power generation beam can only move in the well; that is, it oscillates between the states shown in Figure 7a,b, and the voltage signal output by the power generation beam is shown in Figure 7d. With the increase of external excitation, the power generation beam experiences a sudden snap-through phenomenon and oscillates between the states of moving in the well and moving between the wells (i.e., vibration between the two states shown in Figure 7a,c. The voltage signal output by the oscillating power generation beam is shown in Figure 7e, and due to the impact of sudden snap-through, the peak voltage range jumps from 3500~3900 mV to 11,000~11,500 mV.

**Figure 8:**
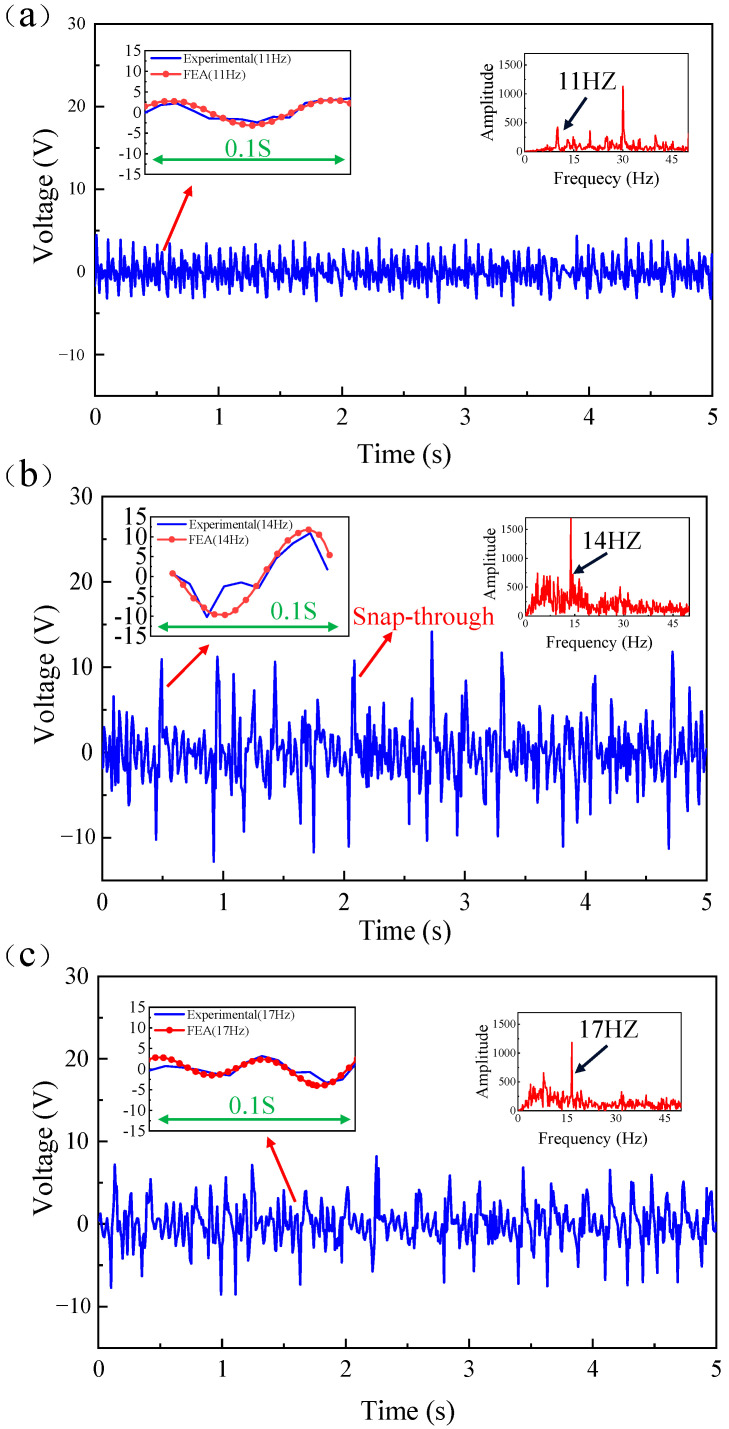
The open-circuit voltage waveforms and the FFT at different excitation frequencies under experimental and FEA: (**a**) 11 Hz, (**b**) 14 Hz, (**c**) 17 Hz.

In order to further evaluate the working performance of the energy harvester in the low-frequency state, we obtained the dynamic response of the energy harvester voltage through experiments, as shown in Figure 8. When the excitation frequency is 11 Hz, the output voltage signal of the generator beam changes with time and the Fast Fourier Transform (FFT), as shown in Figure 8a. It can be found that no snap-through phenomenon occurs at this time, and it moves in the well over a small range, and the output voltage is lower. When the excitation frequency reaches 14 Hz, it can be seen from Figure 8b that the snap-through phenomenon occurs at this time, and the power generation beam transitions between the motion in the well and the motion between the wells, often causing a sharp jump in the output voltage. As the excitation frequency increases to 17 Hz, Figure 8c shows that the peak output voltage increases, but no snap-through phenomenon occurs.

Figure 8 also shows a comparison between experimental tests and finite element simulations at different excitation frequencies. In the upper left corner of the figure, the blue curves correspond to the experimental results, and the red curves with dots correspond to the finite element simulation results. The finite element simulation results are in good agreement with the experimental results. The finite element simulation results are larger than the experimental results, which may be due to the influence of the friction resistance between the proof mass and the steel column during the experimental test. Although petroleum jelly is wiped, the friction resistance still exists, but the finite element simulation does not calculate the friction resistance, so the output voltage is larger than the experimental result.

**Figure 9:**
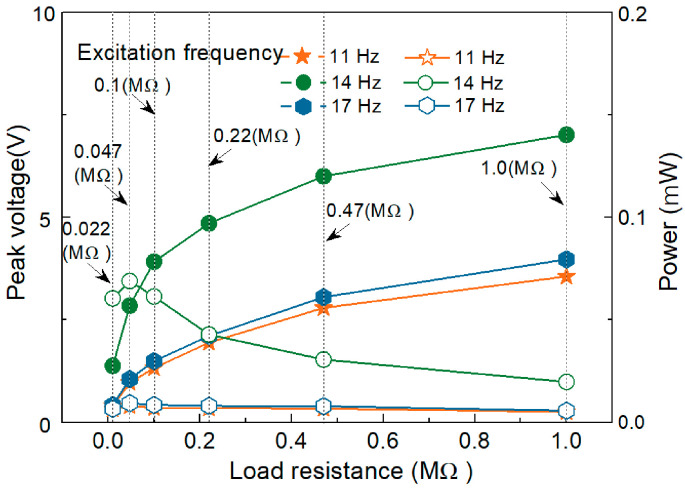
Influence of load resistance on the output power of the harvester.

To further evaluate the performance of the energy harvester proposed in this paper, the output voltage and power under different external load resistors were measured experimentally, as shown in Figure 9. The solid curves in the figure correspond to the average output power (right axis), and the dotted curves correspond to the output voltage signal (left axis). The external load resistance has a great influence on the output power and voltage of the energy harvester. As shown in Figure 9, with the external load resistance increasing from 10 KΩ to 1 MΩ, the output voltage first increases sharply and then increases slowly. This is because the optimal load resistance of the piezoelectric energy harvester is mainly determined by the natural frequency of the structure and the capacitance of the piezoelectric film. The average power $P_{avg}$ is calculated as $P_{avg}$=$\frac{U_{RMS}^{2}}{R}$, where $U_{RMS}$=$\sqrt{\frac{1}{T_{2}-T_{1}}\int_{T_{1}}^{T_{2}}U^{2}dt}$ denotes the root mean square (*RMS*) voltage ($U_{RMS}$), where U is the voltage and R is the resistance. As the external load resistance continues to increase, the output power first increases and then decreases. When the external load resistance is 47 kΩ, the output power reaches a maximum.

A comparison with the harvesters in the literature is provided in Table 1. Although the output power and power density of Huguet et al. and Yi et al. are relatively high, these values are obtained at relatively high acceleration and central frequency levels, which are much higher than those in this work. This work enhances the performance in the low-frequency operating bandwidth (centered at 14 Hz) and achieves an output power of 73 μW, further illustrating the advantages of capturing low-frequency vibration in this work.

## 5. Conclusions

In summary, inspired by the fluttering wings of seagulls during flight, we propose a low-cost, magnet-free, bistable piezoelectric energy harvester to obtain energy from low-frequency vibration and convert it into electrical energy and reduce stress concentration. Moreover, through finite element simulation and experimental tests, the bio-inspired bistable piezoelectric energy harvester is comprehensively studied to evaluate its ability to obtain energy from broadband vibration. The following are the outcomes of this study:

1. When the height of the power generation beam in the bio-inspired bistable energy is 5 mm and the mass of the proof mass is 10 g at the resonant frequency (14 Hz), the power generation beam can break through the potential energy trap and snap-through, switching back and forth between the two stable states, like a seagull flying up and down two stages.
2. The stresses of the seagull-inspired beam proposed in this paper vary continuously with the up-and-down motion of the proof mass block and can well relieve the stress concentration and thus improve its working life. In addition, the peak stress of the seagull-inspired beam is 32.34% lower than that of the parabolic (bow-shaped) beam, indicating that the seagull-inspired beam has huge potential to improve material fatigue failure.
3. When the external resistance of the circuit is 47 kΩ, this model has an open-circuit peak voltage of 11.5 V and a maximum output power of 73 μW.

## Figures and Tables

**Table 1:** Performance comparison of a broadband piezoelectric energy harvester in the papers.

References	Operation Mechanism	Piezomaterial Volume (mm^3^)	Central Frequency (Hz)	Accel. (g)	Power (μW)	Power Density (mW/cm^3^)
Huguet et al. [28]	Bistable and subharmonic	28 × 10 × 0.1	120	0.51	269	9.6
Chen et al. [32]	Buckling	20 × 10 × 0.3	8.2	5	12.2	0.20
Yi et al. [6]	Bi-stability	16 × 5 × 0.05	105.3	2	600	150
Fu et al. [30]	Buckling Plucking	26.5 × 1.5 × 0.2 26.5 × 1.5 × 0.2	23 24.5	0.25 0.08	10.6 5.2	1.34 0.66
This work	Bi-stability	30 × 16 × 0.1	14	N/A	73	1.52

## Data Availability

The introduction data supporting this manuscript are from previously reported studies and datasets, which have been cited. The processed data are available from the corresponding author upon request. The raw test data used to support the findings of this study are available from the corresponding author upon request.
